# Psychotrauma and effective treatment of post-traumatic stress disorder in soldiers and peacekeepers

**DOI:** 10.1186/1745-6673-4-21

**Published:** 2009-07-30

**Authors:** Karin Vitzthum, Stefanie Mache, Ricarda Joachim, David Quarcoo, David A Groneberg

**Affiliations:** 1 Institute of Occupational Medicine, Charité-Universitätsmedizin Berlin, Free University Berlin and Humboldt-University Berlin, Thielallee 69-73, D-14195 Berlin, Germany; 2 Department of Respiratory Medicine, Hanover Medical School, Carl-Neuberg-Straße 1, 30625 Hanover, Germany; 3 Center of Occupational Medicine, Charité-Universitätsmedizin Berlin, Free University Berlin and Humboldt-University Berlin, Augustenburger Platz 1, D-13353 Berlin, Germany

## Abstract

Psychotrauma occurs as a result to a traumatic event, which may involve witnessing someone's actual death or personally experiencing serious physical injury, assault, rape and sexual abuse, being held as a hostage, or a threat to physical or psychological integrity. Post-traumatic stress disorder (PTSD) is an anxiety disorder and was defined in the past as railway spine, traumatic war neurosis, stress syndrome, shell shock, battle fatigue, combat fatigue, or post-traumatic stress syndrome (PTSS). If untreated, post-traumatic stress disorder can impair relationships of those affected and strain their families and society. Deployed soldiers are especially at a high risk to be affected by PTSD but often receive inadequate treatment. Reviews to date have focused only on a single type of treatment or groups of soldiers from only one country. The aim of the current review was to evaluate characteristics of therapeutic methods used internationally to treat male soldiers' PTSD after peacekeeping operations in South Eastern Europe and the Gulf wars.

This systematic literature review returned results pertaining to the symptoms, diagnosis, timing and effectiveness of treatment. Sample groups and controls were relatively small and, therefore, the results lack generalizability. Further research is needed to understand the influence and unique psychological requirements of each specific military operation on the internationally deployed soldiers.

## Introduction

**Figure 1:**
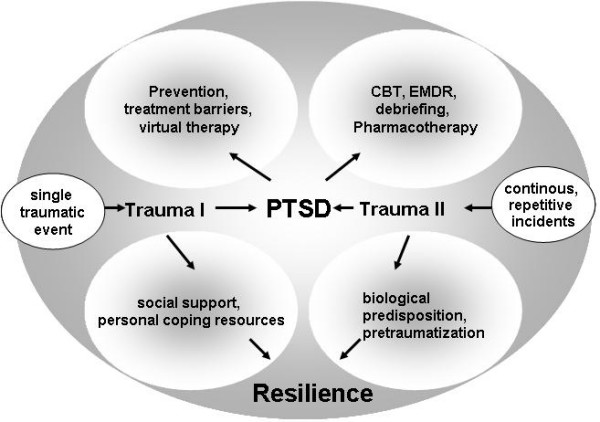
**Model of traumatizing processes and interventions**.

Traumatic events can cause psychological trauma (figure 1). These traumatic events may be single (Type I Traumata), continuous, or repetitive incidents (Type II Traumata) that render inadequate one's ability to cope with the resulting feelings [1]. The inability to cope may become apparent weeks to years after the traumatic experience. The traumatic event may involve witnessing someone's actual death or personally experiencing serious physical injury, rape and sexual abuse, being held hostage, or a threat to physical or mental stability. This is especially true for traumata during childhood. Physical harm, though often a component of the traumatic event, is not mandatory for the development of psychological traumata [2]. Individuals subjected to prolonged periods of extreme poverty or verbal abuse may also be traumatized as a result. Natural disasters, such as earthquakes and volcanic eruptions, and other catastrophes, such as mass violence and military service during war and peacekeeping missions can also cause psychological trauma. This paper focuses on PTSD as a result of male military service during gulf wars and peacekeeping operations in South Eastern Europe. We excluded female and child soldiers and Vietnam veterans explicitly from this review to ensure the highest level of comparability.

Post-traumatic stress disorder (PTSD) is classified as an anxiety disorder (ICD-10 F 43,1, DSM IV 309.81), to develop characteristic symptoms after traumatic exposure and to constantly avoid triggers to revive the traumatic event [1,2]; historically PTSD has also been called railway spine, stress syndrome, shell shock, combat or battle fatigue, traumatic war neurosis, or post-traumatic stress syndrome (PTSS) [3]. Reactions to and symptoms of trauma can be manifold; although most people experience trauma in the course of a lifetime, only 8% of them develop PTSD [4]. Social Support after deployment in Iraq was shown to be a preventive factor even after combat exposure in a recently published study by Pietrzak et al. [5]. Vulnerability to PTSD presumably stems from an interaction between mental and biological predisposition, traumatic experiences during early childhood and severity of the trauma [6-10]. Symptoms may include pessimistic cognitive schemas [11], uncontrollable flashbacks and nightmares, avoidance of triggers associated with the trauma, increased arousal levels, sleeping problems, anger, increased agitation, and increased substance abuse [12]. Per definition, symptoms, like increased arousal, last longer than one to six months in PTSD and can significantly impair one's functioning both personally and professionally [1,2].

### Soldiers and PTSD

Due to greater exposure to combat, members of the Marines and Army are generally believed to be at higher risk for PTSD than members of the Air Force and Navy [13]. If left untreated, PTSD can negatively affect sufferers' behavior and damage their romantic relationships, their families, and involvement in society [14,15].

Officially, the armed services of all the countries in this study offer mental health care services to soldiers; however, many soldiers are hesitant to seek treatment. Many conceal their condition for fear of retribution in the form of intolerance, stigmatization and job loss [16] despite evidence that treatments such as time-limited psychotherapy can achieve sustained improvement in psychosocial functioning and reduced severity of psychiatric and stress-related symptoms [17].

Reviews have largely focused only on specific treatment methods or patient groups; however, soldiers serving in international peacekeeping missions that involve multinational cooperation receive different treatments in their home countries. Therefore, the present review summarizes recently published data on the association of psychotrauma with different psychological treatment concepts. The objective of the current review was to analyze characteristics of therapeutic methods used to treat soldiers deployed to peacekeeping operations and the Gulf wars. Furthermore, we sought to track the latest developments in the field of trauma research and to identify mental structures in this patient group.

## Methods

Standardized keyword searches in PubMed (55 items) and Psycinfo (153 items) were performed using the terms "ptsd treatment" and/or "soldiers" and publication types (date: 2009-03-04, review: July 2009). Approximately 200 articles were analyzed for study design, content and relevance. We excluded 43 reviews from the initial findings. 165 articles were retained and further analyzed in terms of appropriateness, excluding those that did not pertain to the specific issues of this study or its test subjects. During systematic scanning for content and study design we also looked through related articles. Fortytwo publications were included in our final analysis, the majority of which were published between 1992 and 2009.

## Results

### Prevention

Military conflicts are often associated with significant prolonged mental illness. One way of treating disorders is to prevent their onset. However, little is known about the role of prevention in PTSD, although some promising results from "combat stress control units" are available [18,19]. Potential preventive interventions may be classified into three categories: primary, secondary and tertiary [13]. Primary prevention includes the deployment selection process and coaching of soldiers prior to exposure to potentially traumatizing events [20]. Secondary prevention includes several short and highly effective psychological methods (i.e. psychological debriefing) used immediately after traumatizing life events. Tertiary interventions include various types of professional treatments [13].

One hundred and six British soldiers participated in an Operational Stress Training Package prior to their deployment. A randomly selected group of these soldiers also took part in additional post-operational psychological debriefing (PD). Overall rates of PTSD and other psychopathologies were low among these soldiers. Furthermore, lower levels of alcoholism were reported among those who received the Operational Stress Training Package than in the control group [21].

Results from a controlled, non-randomized American study [22] using similar methods were less promising. Differences in mental health outcomes between the parallel study groups were not significant post-deployment. Furthermore, there was no evidence that stress briefing prior to deployment led to reduced psychological distress afterward.

### Barriers to treatment

A recurring theme in the literature is that of the barriers facing those who need treatment. Soldiers tend to be stigmatized for undergoing psychotherapeutic treatment. A few studies were found regarding soldiers who expressed urgent need for mental therapy, but do not seek appropriate treatment [16,17].

Maguen and Litz surveyed 203 active duty peacekeepers before and after their deployment to the Balkans to ascertain peacekeepers' symptoms of stress and their attitudes toward seeking mental health care after returning from peacekeeping missions [23]. Sixty-five peacekeepers were examined pre- and post-deployment. Upon return from Bosnia and Kosovo, between 5% and 9% of servicemen expressed a need for treatment of anger or hostility management, depression, or deployment-related stress. The biggest obstacle between soldiers and treatment was concern about the individual financial burden of treatment. Presence of PTSD symptoms before and after deployment were the most significant predictors of deterred psychotherapy among peacekeepers in Kosovo. Soldiers reported a number of mental health care needs and obstacles that would prevent them from receiving care. Peacekeepers needing the highest levels of care, reported the most difficulties receiving treatment [23].

Similar results were found for American soldiers who served in Iraq and Afghanistan [24,25]. The fear of being stigmatized and endangering their careers deter servicemen from accepting care, even if they recognize their mental health needs. The study by Hoge et al. [25] sought to establish the prevalence of major depression, generalized anxiety and PTSD among 6,201 soldiers who served in four U.S. combat infantry units. Less than 50% of the affected U.S. combat infantry personnel consented to psychotherapy [24,25].

### "Do it yourself" therapy and family coping strategies

There are a few guidebooks, which intend to teach members of the armed services to treat themselves following deployment. The book "Courage after fire: Coping strategies for troops returning from Iraq and Afghanistan and their families" by Keith Armstrong, Suzanne Best, and Paula Domenici serves as a handbook for soldiers (and their affiliates) who have been deployed and feel affected by PTSD [26]. This book includes self-help materials based on cognitive-behavioral treatment (CBT). The authors focus on healthy and self-healing human structures, like inner strength and resiliency. They emphasize that psychological symptoms upon return are normal and transient and, moreover, that only a few soldiers will eventually develop a post-traumatic stress disorder (PTSD). Self-help information for spouses and other family members is also available [15,27], since similar burdens were found for military spouses – but in contrast, they are less worried about stigmatization and more willing to seek professional support [28].

To date there has been little research and clinical attention given to families of affected soldiers [29]. A large Dutch study by Dirkzwager et al. [30] examined secondary traumatization of deployed peacekeepers' partners and parents (including more than 700 spouses and 330 parents). Partners of servicemen with symptoms of PTSD reported more sleeping and somatic problems and less positive social support compared with partners of servicemen with no PTSD symptoms. In addition, these partners rated their matrimonial satisfaction lower. Differences in parents' responses were not significant. Thus, peacekeepers' symptoms affected their partner's situation in many ways. Systemic strategies for treating PTSD should be pursued, considering these results and those of American and Canadian studies [30-33].

### Virtual therapy

One of the latest developments in treating PTSD involves "virtual therapy". This method can be utilized for both preventive and therapeutic purposes. Within this therapeutic process, soldiers are exposed to computer-animated scenarios set in their region of deployment. These animations emulate realistic situations that soldiers may experience in the field and allow them to train their responses or revive their memories. Initial studies with Vietnam veterans were auspicious [34].

Case studies present preliminary results of the preventive use of virtual reality exposure (VRE) therapy to treat Operation Iraqi Freedom veterans suffering PTSD ("virtual Iraq") [35-39]. Responding to unpredictable threats, surviving serious injury and maintaining a constant vigilant state were found to be significant risk factors for the development of PTSD. Therefore, timely and effective interventions, such as VRE, should be developed and appropriately introduced to military personnel. Veterans, who underwent brief VRE treatment, reported improved PTSD symptoms and reduced psychological distress post-treatment (compared with pre-treatment reports) [35-39].

The aim of another study was to increase the efficacy of exposure therapy through the implementation of virtual reality (VR) and to investigate how realistic the VRE portrayal of Iraq is based on soldiers' evaluations [40]. Eighty-six percent of soldiers rated the overall realism of the simulated convoy and city environment from adequate to excellent. Thus, VR Iraq portrays a realistic context, in which VR exposure therapy can be administered. Further follow-up evaluations are needed to assess the outcomes of VR exposure therapy for deployed soldiers and veterans suffering from PTSD [40].

### Non-specific or combined treatments

Various approaches, such as group therapy, prolonged exposure to threatening environments (to purge painful emotions), cognitive processing therapy and nontraditional treatments, such as acupuncture and hypnosis, have been used to treat PTSD. Some scientific support of the efficacy of these approaches has been documented [41,42].

New approaches in mental health care for potentially at-risk US soldiers include universal primary care for PTSD and depression screening and the inclusion of a "care facilitator" to ensure care is continuous [43]. Thirty specially trained nurses and physicians collaborated on the treatment process, which included follow-up care, symptom monitoring, and treatment adjustment, to enhance the interface between primary care and mental health care. After six weeks, roughly 70 patients (out of the more than 4,000 patients originally screened), who participated in collaborative care, experienced clinically significant improvement in PTSD symptoms [43].

Gould, Greenberg and Hetherton examined "trauma risk management" (TRiM), a psycho-educational management program developed by the UK Royal Navy (RN) to treat PTSD. Through adjustments in servicemen's attitudes toward PTSD, TRiM helps patients reduce stress and teaches servicemen to identify at-risk coworkers and refer them to early intervention programs. TRiM training significantly improved psychological outcomes [44].

Litz et al. [45] report the results of the 8-week randomized, controlled proof-of-concept trial comparing a new therapist-assisted, self-management cognitive behavior therapy with internet-based supportive counseling for post-traumatic stress disorder (PTSD). Both treatment groups had roughly 20 participants. Greater reductions in PTSD, depression, and anxiety scores at 6 months were observed in the self-management cognitive behavior therapy group. Six months after the end of treatment, one third of those who completed self-management cognitive behavior therapy reached states of high-end functioning [45].

Fortunato et al. report "little miracles" among veterans who have been treated holistically since May 2008 [46]; unfortunately, they have not yet published scientific studies about their program. One third of the 37 soldiers who completed the treatment successfully could return to their units, even after going through severe catatonic stages. Therapy included 35 hours of treatment per week with daily psychotherapy, group therapy and integrative medicine. In general, soldiers were treated with lower doses of medications and unconventional techniques like medical massage, yoga, tai chi, Qi Gong and Reiki. Other therapeutic forms of exercise included water polo and walking at least 10,000 steps a day (with a daily 45-minute "power walk"). Excursions to crowded and noisy places like shopping malls taught patients to regulate their arousal levels. Learning activities could help create new brain cells to counteract the damage done by elevated stress hormone levels during the deployment. "Rehearsal therapy" helped participants come to terms with their most painful experiences and decrease their emotional involvement [46].

Research results for five types of group therapies (i.e. group based exposure therapy, support, psychodynamic, Imagery rehearsal therapy and CBT-focus groups) are available. In order to determine which type of group therapy provides the greatest amount of both short-term and long-term symptom reduction, more research is needed to compare outcomes of the most common forms of group therapy [47-51].

A current large Australian study (n = 4339) suggests that it is more important to consider PTSD severity than type of program regarding the outcome. Soldiers with milder symptoms profit most by moderate-intensity programs [52]. Brown et al. suggested "phase-oriented treatment as the standard of care" since more than a decade [53].

A study with former Croatian soldiers revealed that treatment, which included psychotherapy, had advantages over pharmacological treatment without psychotherapy [54,55]. Vice versa it can be said that "medication does not treat the causes of PTSD, but can relieve secondary symptoms" [56].

### Debriefing

Debriefing intervention is a short-term therapeutic technique that demonstrated good results for PTSD in the wake of natural disasters. Some recent studies have cast doubt upon positive effects of debriefing on preventing the incidence of PTSD. Its role in PTSD treatment remains controversial. One study described the effects of group debriefing among British soldiers returning from peacekeeping operations in Bosnia. Psychological debriefing had a significant effect in reducing alcohol abuse within the study group. The findings of this study suggest although further research is needed, it is too early to assume that debriefing is ineffective in PTSD treatment [57].

Smith and Brady conducted a study of a seven-step critical incident stress debriefing method (CISD). Two U.S. army military police officers (MPs) and 11 Iraqi detainees, who witnessed the untimely death of a fellow detainee, comprised the study group. The primary goal of this treatment was to decrease the impact that traumatic events had on individuals and prevent the onset of post-traumatic stress disorder. Additionally, this method allowed MPs and detainees to confront and refute preconceived notions about each other and encourage future mutual collaboration [58].

### Eye Movement Desensitization and Reprocessing

Eye Movement Desensitization and reprocessing therapy combines effective conventional psychotherapeutic techniques with treatments that stimulate the brain. It focuses on the past experiences that have set the foundation for pathology, the present situations that trigger emotional dysfunctions, one's beliefs and sensations, and the experiences required to positively affect mental health and alter future adaptive behaviors. The most characteristic procedural element is bilateral stimulation of the central nervous system through eye movements, tones or taps. During the reprocessing phases, patients momentarily dwell on past memories, current triggers, or anticipated future experiences while simultaneously concentrating on external stimuli. During these reprocessing procedures, patients report experiencing new insights, changes in their memories, or new associations between these different aspects [59,60]. Positive short term effects of EMDR were reported for Vietnam veterans [61,62].

A German study [48] compared 89 soldiers returning from peacekeeping missions who were treated with either EMDR or with relaxation exercises. The authors report that inpatient treatment with eye movement desensitization and reprocessing significantly improved the course of patients' PTSD. Interestingly, the Impact of Event Scale demonstrated significantly poorer long-term outcomes for patients who had come face-to-face with death during the traumatic event [48].

## Discussion/Conclusion

Despite media hype and an increased public interest in post-traumatic stress disorder, this review of empirical PTSD research is the first of its kind. We focused on how different military and public health systems manage the treatment of soldiers and peacekeepers suffering from PTSD after returning home from deployment. Scientific literature on psychotherapy for PTSD is scant and might be an indirect consequence of soldiers' fear of stigmatization.

Our findings suggest that post-traumatic psychological treatment can have short- and long-term advantages for affected army members, although study results are often difficult to compare due to small sample numbers, different types of deployment, different methodological approaches and the variable severity of traumatization [29,48].

Evidence supporting the effectiveness of stress debriefing is inconclusive and its indication in the treatment of PTSD remains controversial [22]. Barriers to mental health care, such as stigmatization, still prevent affected soldiers from seeking and receiving needed treatment [16,17]. Future strategies for PTSD may lie in the field of prevention and in investigating successful coping mechanisms among resilient servicemen [48]. Especially newer techniques, such as "virtual therapy," could offer an inexpensive alternative treatment with potentially global application. The easy accessibility of such techniques may help increase treatment participation by reducing soldiers' fear of being stigmatized [35,36]. We did not find systematic studies, which examined a pharmacological treatment combined with psychotherapy [46]. This could obscure further resources of treatment improvement. Future studies should concentrate on prevalence and baseline data related to traumatic severity and type, combat exposure and its specific co-morbid symptoms, such as substance abuse [1,29,33,58].

Results of national studies cannot be generalized. Influences of multinational characteristics of disease management need to be investigated in more detail. Treatment of mental conditions is not only influenced by the general organization of a healthcare system, but also affected by the society, in which soldiers live, and by the current values within that society [54,55].

We expect new insights on treatment success, since researchers are more aware of PTSD in general and affected soldiers in particular. Servicemen are more likely to be treated immediately after traumatization nowadays instead of being examined years after deployment in Vietnam [25].

Although significant advances have been made, further investigation is needed in several areas of PTSD research to understand the impact and unique psychological requirements of military operations on the internationally deployed soldiers [48]. Further research and treatment efforts should focus more on multinational cooperation and multimodal approaches to increase the efficacy of PTSD treatment.

## Competing interests

The authors declare that they have no competing interests.

## Authors' contributions

All authors have read and approved the final version and the manuscript has not been funded, submitted or published anywhere else. KV, SM and DAG designed the study. KV and RJ performed the search routines. KV, DQ and DAG performed pilot data search routines and analysis. 
